# Histopathological Examination of the Mucosal Effects of Obstetric Gel on Vaginal Wound Healing in an Incision-Inflicted Rat Model

**DOI:** 10.7759/cureus.18254

**Published:** 2021-09-24

**Authors:** Pınar Kırıcı, Selçuk Kaplan, Bilge Aydin Turk, Ebru Annac

**Affiliations:** 1 Obstetrics and Gynaecology, Adiyaman University, Adıyaman, TUR; 2 Obstetrics and Gynaecology, Adiyaman University Faculty of Medicine, Adıyaman, TUR; 3 Pathology, Adiyaman University Faculty of Medicine, Adıyaman, TUR; 4 Histology and Embryology, Adiyaman University Faculty of Medicine, Adıyaman, TUR

**Keywords:** fibrillin-1, rat, episiotomy, wound healing, obstetric gel

## Abstract

Background and objective

The present study intended to investigate the histopathological efficacy of obstetric gels on the healing of vaginal lacerations in rats. To the best of our knowledge, this is the first such study.

Materials and methods

Twenty-one female Wistar albino rats were divided into three groups, comprising seven animals per group. The first group (group 1) was the control group, the second (group 2) was the polyvinyl iodine (PI) group, and the third group (group 3) was the obstetric gel (OG) group. In all three groups, a vaginal incision was made with a No. 10 scalpel, and the incision site was sutured with a 3-0 Vicryl suture. In the control group, the incision site was left for routine healing. The incision site was washed with PI in the PI group and with OG in the OG group. After 15 days, vaginal tissues were obtained from all three groups for histopathological examination. In addition, immunohistochemistry staining was performed using caspase 3 and fibrillin 1 antibodies.

Results

There was no significant difference between the groups in terms of congestion, vascular proliferation, and inflammation stages in the examinations performed on the vaginal wall. However, the amount of collagen and elastic fibers increased during the remodeling and fibrosis phase, and the fibrillin 1 score increased in immunohistochemistry staining (p < 0.001).

Conclusion

It has been shown in rat vaginal tissue that obstetric gels do not have negative effects on wound healing; however, they contribute to wound healing by positively affecting the fibrosis stage.

## Introduction

The second stage of labor is the period from full dilation of the cervix to birth and is the shortest stage of labor [1]. A prolonged second stage of labor is associated with an increased risk of maternal complications, such as perineal trauma, chorioamnionitis, and bleeding, all of which require additional medical interventions. A history of difficult delivery due to a prolonged second stage of labor is one of the most important reasons for vaginal tissue lacerations [2-3].

Lacerations that occur in the vagina cause maternal morbidity in the postpartum period [4]. Shortening the second stage of labor might help preserve the integrity of the perineum and prevent possible negative maternal and neonatal consequences. Episiotomy, use of forceps/vacuum, or positional methods, such as lateral positioning, are used to shorten the second stage of labor, minimize the development of perineal trauma, and protect the integrity of the perineum [5]. Polyvinyl iodine, lavender oil, and aloe vera gel have been used as lubricants in various studies to facilitate vaginal delivery [6-7]. There is no standard procedure for the routine use of such lubricants in vaginal delivery [8].

The effectiveness of vaginal obstetric gels (OG) to shorten the second stage of labor has been investigated in some studies [9-10]. It is felt that to shorten the second stage of labor, OG can be used to reduce the friction forces affecting vaginal delivery. However, data on the effectiveness of OG are insufficient.

Further, there is no study showing the effectiveness of OG on wound healing in vaginal lacerations that occur during vaginal delivery. The present study is intended to investigate the histopathological efficacy of the OG on the healing of vaginal lacerations.

To the best of our knowledge, this study is the first to investigate the histopathological efficacy of OG on wound healing in vaginal lacerations in rats.

## Materials and methods

Ethical approval

Experiments in this study were conducted in accordance with the National Institutes of Health animal research guidelines and approved by our animal ethics committee (Date: 04.06.2020, Ethics committee no: 2020/017).

Animals and experimental protocol

Twenty-one female Wistar albino rats, weighing 250-280 g, were divided into three groups comprising seven animals in each group. The first group (group 1) was the control group, the second (group 2) was the polyvinyl iodine (PI) group, and the third group (group 3) was the OG group.

Experimental protocol

Before the experiment protocol, 50 mg/kg ketamine and 10 mg/kg xylazine were administered intraperitoneally. In the control group (group 1), after anesthesia, a vaginal incision was made with a No. 10 scalpel blade. The incision site was sutured with a 3-0 Vicryl suture. In the PI group (group 2), after anesthesia, a vaginal incision was made with a No. 10 scalpel blade. The incision site was sutured with a 3-0 Vicryl suture. The incision was washed with PI. In the OG group (group 3), after anesthesia, a vaginal incision was made with a No. 10 scalpel blade. The incision site was sutured with a 3-0 Vicryl suture. The incision site was washed with OG. An OG containing propylene glycol, carbomer, hydroxyethylcellulose, and deionized water was used. After 15 days, vaginal tissues were obtained from all three groups for histopathological examination. After the surgical procedure, all rats were sacrificed. Histopathological examination was performed in the Departments of Pathology and Histology, Adıyaman University Medical Faculty Hospital.

Histopathological evaluation

Sections of 5 µm thick vaginal tissue obtained from paraffin blocks using the Thermo Shandon Finesse ME microtome device (Thermo Fisher Scientific, Cheshire, UK) were stained using the hematoxylin-eosin, Masson’s trichrome, Verhoeff-Van Gieson, and toluidine blue staining methods and examined under a light microscope [11-13]. In addition, immunostaining was performed using caspase 3 and fibrillin 1 antibodies. Histopathological evaluations were performed by examining the tissue layers. For the evaluations, Carl Zeiss Axiocam brand ERc5 model (Carl Zeiss Microscopy GmbH 07745 Jena, Germany) digital camera attachment microscope device was used.

Hematoxylin-Eosin Staining

The excised vaginal tissue was fixed with 10% formalin solution for histopathological examination. Approximately, 5 µm thick sections were obtained from the formalin-fixed vaginal tissue. Samples were stained with hematoxylin-eosin and examined under a light microscope.

Masson’s Trichrome Staining

Vaginal tissues were evaluated semi-quantitatively for fibrosis. First, 6 µm thick sections were deparaffinized and passed through alcohol batches. Then the sections were treated with Weigert´s iron hematoxylin and later incubated in acid fuchsin. It was then treated with phosphomolybdic acid and finally stained with methyl blue. Collagen fibers were stained blue as a result of the staining.

Verhoeff-Van Gieson Staining

Transverse sections of 4 µm thickness were obtained from the tissues that were stained with a Verhoeff's-Van Gieson staining block and placed on adhesive slides. We utilized Verhoeff's staining method to reveal elastic fibers and the Van Gieson staining method to reveal smooth muscle tissue in the tunica media.

Coloring Toluidine

The metachromatic substances appear red-pink or purple, and the nuclei and other components appear blue with toluidine blue staining. It was used to identify mast cells due to the effect of heparin in cytoplasmic granules and to stain proteoglycans in tissues such as cartilage [13].

Immunohistochemical evaluation

For fibrillin 1 and caspase 3, the avidin-biotin-peroxidase (ABC) complex was applied under minor modifications in the tissue [14]. With this method, 5 µm thick sections were obtained from the blocked tissues and they were deparaffinized. The primary antibody fibrillin 1 (Rabbit polyclonal Fibrillin-1, Rabbit Polyclonal bs-1157R Bioss Antibodies Massachusetts) and caspase 3 (Rabbit polyclonal, Caspase-3 Abcam, ab2302, London, UK) were used. Positive and negative controls were made as per the manufacturer’s recommendations. After the application of AEC Chromogen, it was stained with Mayer hematoxylin and examined under a light microscope. The preparations thus prepared were evaluated using the Carl Zeiss Axiocam brand ERc5 model (Carl Zeiss Microscopy GmbH 07745 Jena, Germany) under the microscope. The degree of staining was determined as 0: not present, +0.5: very low, +1: low, +2: medium, +3: severe [15].

Wound healing was evaluated in five stages: granulation, hemostasis, inflammation, proliferation, and remodeling [16]. Vacuolization in the epithelium, keratohyalin, and epithelial cells was evaluated in the granulation phase; vascular congestion in the hemostasis phase; mast cells, and polymorphonuclear cell infiltration in the inflammation phase; and elastic fibers, collagen amount, and fibrillin 1 immunohistochemistry staining in the proliferation and remodeling phases. These parameters were separated semi-quantitatively as mild (0%-33%), medium (34%-66%), and severe (67%-100%) and scored as mild: +1, moderate: +2, and severe: +3 for immunohistochemical staining [17].

Statistical analysis

Statistical analyses were performed using IBM SPSS Statistics 22.0 (IBM Corp, Armonk, NY). The Shapiro-Wilk test, histograms, and Q-Q graph tests were used to determine whether the data were normally distributed. Descriptive statistics are defined as median ± standard deviation and median (minimum and maximum values). The chi-square test (Pearson's chi-square and Pearson's exact chi-square tests) was used to compare the proportions between groups. One-way analysis of variance (ANOVA) was used to compare the means between the groups, and the post-hoc test was used to find out which group generated significance.

## Results

Histopathological examination

Mucous Layer

**Figure 1:**
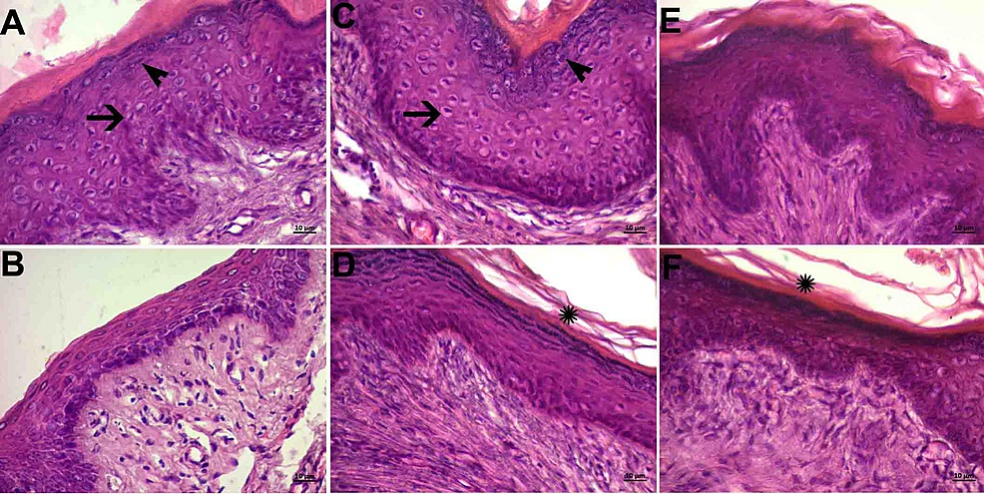
Histopathological examination of the mucous layer A and B: Control group; C and D: PI group; D and E: Histological images belonging to the OG group (hematoxylin-eosin staining and objective magnification of x40). Epithelium of the multi-layered flat type (A, C, and E). Regions where the thickness decreased due to compression (B, D, and F). Black arrow, vacuolization in cells; black arrowhead, keratohyalin granules in superficial cells; star, cornfield epithelial cells OG: obstetric gel; PI: polyvinyl iodine

There were many transverse folds and folds in the mucosal layer in all the groups. The epithelium appeared to be the multi-layered flat type (Figure 1, panels A, C, and E). There were areas of locally decreased epithelial thickness (Figure 1, panels B, D, and F). In the control and PI groups, vacuolization was detected in the cells and keratohyalin granules on the cell surface (Figure 1, panels A and C). Cornfield epithelial cells were observed (Figure 1, panels D and F).

**Figure 2:**
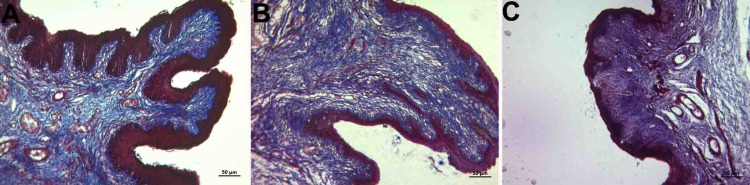
Histopathological examination of the lamina propria A: control group; B: PI group; C: Histological images belonging to the OG group (Masson trichrome staining and x10 objective magnification). Dense collagen fiber dominance was observed in the connective tissue. OG: obstetric gel; PI: polyvinyl iodine

**Figure 3:**
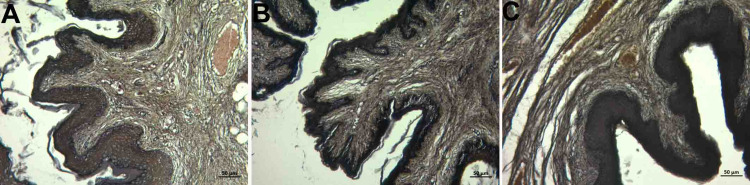
Histopathological examination of the lamina propria A: control group; B: PI group; C: Histological images belonging to the OG group (Verhoeff's Van Gieson staining and x10 objective magnification) The elastic fiber was dense in connective tissue in all groups. OG: obstetric gel; PI: polyvinyl iodine

**Figure 4:**
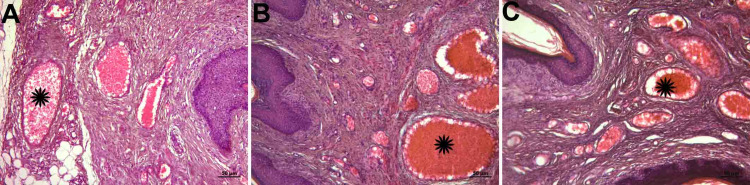
Histopathological examination of vessels A: control group; B: PI group; C: histological images belonging to the OG group (hematoxylin-eosin staining and objective magnification of x10); Star; congestion Signs of congestion in the vessels were detected in all groups. OG: obstetric gel; PI: polyvinyl iodine

Abundant collagen and elastic fibers were observed in the lamina propria, which is a tight connective tissue layer (Figure 2, panels A, B, and C; Figure 3, panels A, B, and C). The connective tissue had abundant blood vessels. There were signs of congestion in the vessels in all groups (Figure 4, panels A, B, and C) (Table 1).

**Table 1:** Descriptive analysis of wound healing parameters scores *statistically significant PI: polyvinyl iodine; OG: obstetrics gel

Group		Inflammation	Granulation	Fibrosis	Vascular Congestion	Fibrillin 1
Control	Mean	2.02±0.21	2.01±0.007	2.91±0.07	1.88±0.11	0.47±008
PI	Mean	1.12±0.18*	2.23±0.24	2.11±0.46	2.69±0.62	1.73±0.21
OG	Mean	2.74±0.21	2.53±0.23	2.93±0.63*	1.80±0.37	2.77±0.17*
	p	<0.001	0.108	<0.001	0.139	<0.001

**Figure 5:**
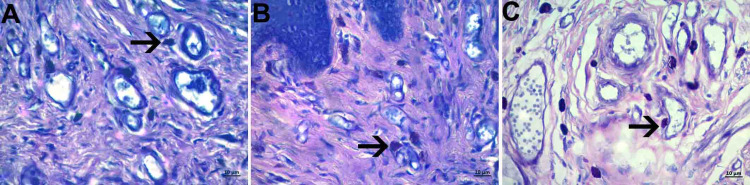
Mast cell density A: control group, B; PI group; C; histological images belonging to the OG group (Toluidine blue staining and objective magnification of x40); black arrow: mast cell It was observed that the mast cell density increased in the gel group. OG: obstetric gel; PI: polyvinyl iodine

Mast cell density in the connective tissue areas around the vessel was increased in the OG group when compared with the other groups (Figure 5, panels A, B, and C).

Muscular Layer

**Figure 6:**
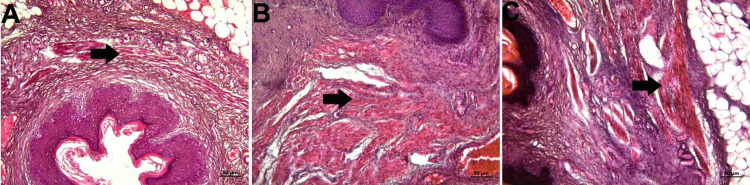
Histopathological examination of the muscular layer A: control group; B: PI group; C: histological images belonging to the OG group (hematoxylin-eosin staining and objective magnification of x10); black arrow; muscle fibers There was no difference in the muscle layer between the groups. OG: obstetric gel; PI: polyvinyl iodine

The muscular layer underneath the lamina propria consisted of smooth muscle fibers that were circular inside and longitudinal outside. The boundaries of the inner and outer regions were not very clear. Muscle fibers were surrounded by connective tissue septa. When the groups were compared, no difference was observed (Figure 6, panels A, B, and C).

Adventitia

**Figure 7:**
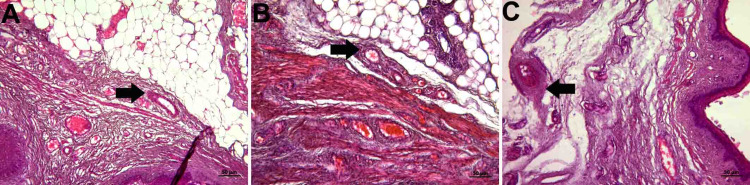
Histopathological examination of the adventitia A: control group; B: PI group; C: histological images belonging to the OG group (hematoxylin-eosin staining and objective magnification of x10); black arrow: adventitia layer There was no difference in the adventitia layer between the groups. OG: obstetric gel; PI: polyvinyl iodine

Adventitia was in the form of a thin connective tissue rich in elastic fibers that connected the vagina with the external structures. There were vascular structures. No difference was detected between the groups (Figure 7, panels A, B, and C).

Immunohistochemical examination

Fibrillin 1 and Caspase 3

**Figure 8:**
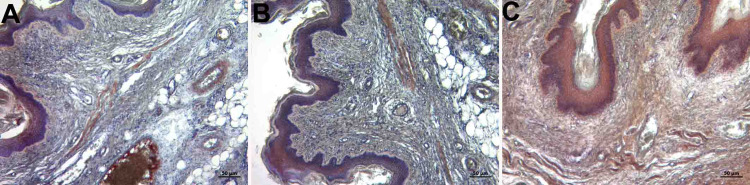
Immunohistochemistry staining with fibrillin 1 A: control group; B; PI group, C: histological images belonging to the OG group (fibrillin 1 immunohistochemistry staining and x10 objective magnification) +0.5: very little in the control and PI groups; +3: severe involvement was observed in the OG group OG: obstetric gel; PI: polyvinyl iodine

**Figure 9:**
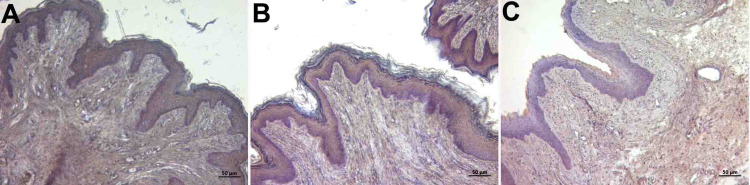
Immunohistochemistry staining with caspase 3 A: control group; B: PI group; C: histological images belonging to the OG group (caspase 3 immunohistochemistry staining and x10 objective magnification) +2: moderate involvement was observed in control, PI, and OG. OG: obstetric gel; PI: polyvinyl iodine

In the immunohistochemical evaluation, while +0.5 (very little) involvement was detected in the control and PI groups in fibrillin 1 staining, +3 (severe) involvement was observed in the OG group (Figure 8, panels A, B, and C). In the caspase 3 immunohistochemistry staining, +2 (moderate) involvement was observed in the groups. There was no significant difference between the groups (Table 1) (Figure 9, panels A, B, and C).

## Discussion

The present study evaluated the effect of OG on the healing of perineal lacerations using multiple histopathological criteria and found that OG contributed to the proliferation and remodeling stages of wound healing.

Episiotomy is a surgical enlargement of the vaginal orifice with a perineal incision performed in the concluding or the second stage of delivery [18]. Although it has been reported that obstetricians should electively perform a surgical incision or episiotomy to reduce the incidence of spontaneous perineal lacerations occurring during delivery, it was demonstrated that the healing of perineal trauma with routine episiotomy was not justified by evidence [19-20]. Although infection, pain, and prolonged wound healing secondary to episiotomy are important complications [21] of the prolonged second phase of labor, perineal lacerations can also occur due to reasons such as a difficult birthing process and a large fetus despite the performance of episiotomy. Many women experience perineal trauma during vaginal delivery. Albers states that the rate of perineal trauma is high during first vaginal deliveries [22]. Perineal injuries increase postpartum morbidity in women and cause psychological trauma.

There are some studies investigating techniques, such as perineal massage and heat compress application, during labor to reduce the rate of perineal lacerations [23-24]. In addition, it was reported in one study that vaginal flora affects neonatal gastrointestinal flora; therefore, OG with microbicidal qualities may cause poor perinatal outcomes [25].

Since the effects of OG on perineal and vaginal episiotomy wounds have not been evaluated and due to the lack of adequate data on this subject in the literature, we performed an episiotomy procedure in rats in the present study and examined the histopathological effects of OG.

To the best of our knowledge, there is no study evaluating the histopathological changes in perineal injury in rats. In the present study, wound healing was evaluated using multifaceted histopathological semiquantitative evaluation methods, and it was found that fibrogenesis was more enhanced in the proliferation and remodeling phases of wound healing in the OG group when compared with other groups.

The process of recovering the integrity of the skin is a complex biological process consisting of wound healing, hemostasis, inflammation, proliferation, and remodeling [26]. Episiotomy incision healing has a similar process. The healing process begins with the incision of the mucosal epithelium and perineal lacerations [27]. Since the angiogenic factor is released during the inflammation phase, vascularization or angiogenesis is also linked with the inflammatory phase. A healthy wound healing should be supported by the development of fibrosis. Fibrosis begins with the collagen synthesis of myofibroblasts following the end of the inflammatory process. Fibrillin is the main component of extracellular matrix (ECM) microfibrils that provide tissue elasticity and plays an important role in the fibrosis stage by providing the formation of elastic fibrogenesis in the tissue [28-29]. Factors affecting this process can lead to an abnormal response [30]. It is thought that practices that could accelerate and regulate the process by affecting the stages of wound healing can prevent complications that may occur during this process.

In our study, the number of mast cells increased in the mucosal layer in the group of rats treated with OG, and the fibrillin 1 score in immunohistochemistry staining was also significantly higher in the OG group than the other groups. This suggests that the OG may be useful in the final stage of wound healing.

## Conclusions

The results of our study showed that OG, used in the second stage of labor due to its lubricating property, did not adversely affect wound healing, and contributed to wound healing by positively affecting the fibrosis stage in rat vaginal tissue. This suggests that OG can be preferred in vaginal delivery not only because of its lubricant feature, but also because it accelerates the late stage of wound healing. However, further studies in large human populations are needed to demonstrate the effectiveness of OG on wound healing.
